# Thoracic limb stride length is associated with cognitive impairment in aging dogs

**DOI:** 10.3389/fvets.2026.1814017

**Published:** 2026-06-25

**Authors:** Shaghayegh Rafatpanah Baigi, Akiya Stywall, Chin Chieh Yang, Alejandra Mondino, Gilad Fefer, Wojciech K. Panek, Katherine E. Simon, Beth C. Case, Margaret E. Gruen, Natasha J. Olby

**Affiliations:** 1 Department of Clinical Sciences, College of Veterinary Medicine, North Carolina State University, Raleigh, NC, United States; 2 Department of Clinical Sciences, Faculty of Veterinary Medicine, Université de Montréal, Montréal, QC, Canada; 3 Department of Clinical Sciences & Advanced Medicine, School of Veterinary Medicine, University of Pennsylvania, Philadelphia, PA, United States

**Keywords:** aging, canine cognitive dysfunction, canine gerontology, gait analysis, mobility, stride length

## Abstract

**Introduction:**

Changes in stride length have been linked to cognitive impairment in humans with dementia. In aging dogs, cognitive decline is accompanied by slower gait speed. However, the relationship between stride length and cognitive decline has not been investigated. This study examined whether height-adjusted stride length is associated with owner-reported cognitive impairment in aging companion dogs.

**Methods:**

Data were collected from a cohort of client-owned senior and geriatric pet dogs, enrolled in the Longitudinal Study of Canine Neuroaging. On-leash gait was recorded on a standardized 5 m walkway, and stride lengths were derived from video analysis and normalized to withers height. Cognitive function was assessed using the Canine Dementia Scale (CADES), and pain was evaluated using the Canine Brief Pain Inventory (CBPI). Associations between height-adjusted stride length, age, CADES, and CBPI scores were examined using linear mixed-effects models.

**Results:**

Eighty-eight dogs were enrolled. Height adjustment reduced the influence of body size on stride length. Thoracic limb height-adjusted stride length decreased significantly with age, whereas pelvic limb stride showed no significant age-related association. Although CADES scores increased with age, their association with stride length persisted after adjustment for age and pain, while age alone was not a significant predictor in the multivariable model. Intra-observer and interobserver reliability for stride length measurements were excellent.

**Conclusion:**

These findings support the use of thoracic limb stride length as an objective, scalable functional mobility measure that reflects changes associated with cognitive decline and may be a useful tool for research and clinical monitoring of aging in dogs.

## 1 Introduction

Mobility decline is a major outcome of aging and a predictor of independence, quality of life, and survival (1, 2). In older adults, walking performance reflects both nervous and musculoskeletal systems, and changes in gait are strongly associated with disability, falls, and mortality (1, 3). Spatial gait characteristics, such as stride length, have been shown to worsen with declining cognition, often independent of overt musculoskeletal disease (4, 5). In individuals with Alzheimer-type dementia, more advanced disease is associated with slower walking speed and shorter stride length (5, 6). One study that compared fallers and non-fallers within dementia severity groups found that stride length variability was a particularly sensitive discriminator, suggesting that impaired gait regulation is linked to cognitive dysfunction rather than musculoskeletal disease alone (6).

Aging companion dogs experience similar changes in mobility, activity levels, and cognitive function (7, 8). Canine Cognitive Dysfunction Syndrome (CCDS) resembles Alzheimer's disease in its pathological changes and clinical manifestations (9, 10). CCDS is characterized by a range of behavioral signs, including disorientation, impaired learning and memory, altered social interactions, anxiety, sleep–wake cycle disturbance, and house soiling (11, 12). In addition to these behavioral changes, owners report physical signs such as unsteadiness, swaying, and falls (8, 13).

Mobility is essential to the quality of life of companion dogs (13). Reduced activity and impaired gait in older dogs are associated with poorer quality of life and increased mortality risk (2, 14). Previous work in aging pet dogs has demonstrated that slower gait speed and reduced activity are associated with advancing age and poorer cognitive status using owner-reported measures (7, 15). However, gait speed alone may be insufficient to capture early or subtle dysfunction, as it does not fully reflect changes in movement quality or functional mobility (13). Measures that reflect gait organization and motor planning, such as stride length may therefore provide additional insight into age-related functional change beyond what is captured by gait speed alone (4).

The relationship between mobility and cognition in aging dogs represents an important area of interest, particularly given the evidence from human studies that spatial gait parameters as simple as stride length reflect cognitive dysfunction. It is currently unknown whether stride length is associated with owner-reported cognitive impairment in aging dogs (4, 6, 7). Addressing this gap is clinically relevant for veterinary medicine, as identifying simple, objective markers of declining function could improve monitoring of aging dogs and inform development of management plans (14, 16).

The aims of this study were firstly to describe the longitudinal changes in thoracic and pelvic limb height-adjusted stride length in aging companion dogs, and secondly to determine whether height-adjusted stride length is associated with owner-reported cognitive decline. We hypothesized that height adjusted stride length would decline with age and correlate with cognitive performance.

## 2 Materials and methods

### 2.1 Study design and population

Data for this study were drawn from the Longitudinal Study of Neuroaging in Dogs, a prospective longitudinal cohort study conducted at the North Carolina State University College of Veterinary Medicine, enrolling client-owned senior and geriatric dogs with a fractional lifespan of ≥0.75 (defined as the proportion of expected lifespan attained), with expected lifespan calculated using height and weight (17). Dogs are evaluated longitudinally across multiple domains, including physical health, cognition, sensory function, mobility, and muscle mass. Detailed descriptions of the overall study design and methodology have been published previously (7, 14, 18).

All study procedures were reviewed and approved by the North Carolina State University Institutional Animal Care and Use Committee (IACUC protocol numbers 18–109, 21–303, and 24–255). Written informed consent was obtained from all owners prior to enrollment.

Dogs were evaluated approximately every 6 months with each 6-month interval defined as a study visit. Each visit consisted of three consecutive study days. On Day 1, dogs underwent complete physical, neurologic, and orthopedic examinations, mobility assessments, functional hearing testing, and laboratory evaluations, including complete blood count, serum biochemistry panel, and urinalysis. On Day 2, dogs completed a standardized cognitive test battery. Day 3 was dedicated to electrophysiologic testing, including brainstem auditory evoked response (BAER) and electroretinography (ERG). Owners were asked to complete several questionnaires at each 6-month time point, including the Canine Dementia Scale (CADES), and Canine Brief Pain Inventory (CBPI). Age (years), sex, breed, weight (kg), and height (cm) were recorded at each visit. Height was measured for each dog in centimeters as the vertical distance from the ground to the withers. Longitudinal data were collected and managed using REDCap (Research Electronic Data Capture), an electronic data capture tool hosted at North Carolina State University (19, 20).

### 2.2 Gait speed assessment and stride length

On-leash gait speed was assessed using a straight, unobstructed 5-m indoor walkway following a standardized operating procedure. Handlers walked the dogs using their own leash, kept slack to minimize handler's influence on speed, allowing the dogs to set the pace. No verbal encouragement, treats, or other external motivation were used.

Each trial included a 3-m acceleration zone before and a 3-m deceleration zone after the timed 5-m walkway allowing dogs to reach and maintain a steady pace. The handler announced the word “start” and began the stopwatch when the dog's first thoracic limb crossed the start line of the 5-m zone. When that same limb crossed the finish line, the handler announced the word “stop” and stopped timing. Time was recorded using a handheld stopwatch.

Each dog completed two on-leash trials (walking toward the room entrance and away from the entrance). Gait speed (m/s) was calculated for each trial by dividing 5-m by the recorded time. Gait speed and stride length were derived from the same standardized 5-m walkway trials. Videos were simultaneously acquired from a fixed position approximately midway along the 5-m zone, with the camera placed at a consistent height and angle and panned smoothly as the dog crossed the walkway. The start and finish lines were visible in the recording allowing accurate step counting. Trials were aborted and repeated if the dog stopped, changed direction, pulled on the leash, turned their head repeatedly, crossed outside the walkway, or was otherwise distracted.

Video recordings from the standardized 5-m walkway trials were reviewed by 2 trained observers. For each pass, the number of steps taken by each limb was counted, including the left thoracic (LT), right thoracic (RT), left pelvic (LP), and right pelvic (RP) limbs. Two passes (one in each direction) were analyzed for each dog.

Stride length (SL) for each limb was calculated by dividing the walkway length (5-m) by the mean number of steps for that limb across the two passes, according to Equation 1. Therefore, step counts were first averaged across passes, and a single stride length value per limb was then derived from this mean.


$$\begin{array}{l} \text{SL}(\text{m})=\;\frac{5}{mean\;step\;count\;across\;two\;passes\;for\;that\;limb} \end{array}$$ (1)

Height-adjusted stride length (SLadj) was calculated by normalizing stride length to body size using Equation 2:


$$\begin{array}{lll} \text{SL}\;\text{adj} & = & \;\frac{\text{SL}}{withers\;height\;\left(cm\right)} \end{array}$$ (2)

For statistical analyses, stride length was summarized as the mean of the left and right height-adjusted stride lengths (mean SLadj), which was defined as the primary dependent variable.

For repeatability assessment, a subset of 7 dogs with 32 measurements underwent repeated scoring by the same rater (intra-observer reliability), and 10 dogs with 20 measurements were independently scored by a second rater (interobserver reliability).

### 2.3 Owner Questionnaires

Cognitive function was assessed using a validated owner-reported instrument: the Canine Dementia Scale (CADES). CADES is a 17-item questionnaire assessing cognitive dysfunction across four behavioral domains (spatial orientation, social interaction, sleep–wake cycles, and house soiling) and provides a continuous measure of cognitive impairment (21). CADES has been validated for use in aging dogs and was completed by owners at each 6-month time point.

To account for potential confounding effects of osteoarthritis pain on gait measures, owners completed the Canine Brief Pain Inventory (CBPI) questionnaire (22, 23). CBPI provides a composite measure of owner-perceived pain severity and pain-related functional interference. Total scores from this clinical metrology instrument were used as covariates in statistical models.

### 2.4 Statistical analysis

Summary data for thoracic (TL) and pelvic limb (PL) SL and height-adjusted SL (SLadj) were generated. The association between SL and SLadj and body weight was evaluated using simple linear regression models to assess the influence of body weight and to confirm the effectiveness of height adjustment in minimizing size-related effects.

To assess the reliability of stride length measurements, both intra-observer and interobserver reliability were evaluated using intraclass correlation coefficients (ICC). Intra-observer reliability was assessed by repeat stride length measurements performed by the same observer at two separate time points, 3 weeks apart. Interobserver reliability was assessed by comparing stride length measurements obtained independently by two observers. ICCs were calculated using a two-way mixed-effects model for intraobserver reliability [ICC (1, 3), consistency] and a two-way random-effects model for interobserver reliability [ICC (1, 2), absolute agreement]. Reliability was interpreted according to commonly used thresholds, with ICC values greater than 0.90 considered indicative of high reliability (24).

The association between mean TL SLadj and mean PL SLadj and age was evaluated using linear mixed-effects models, with dog study ID included as a random effect to account for repeated measurements within dogs. The interaction between thoracic and pelvic limb stride length and age was further evaluated using a mixed-effects model including limb (TL versus PL), age, and their interaction, with dog study ID as a random effect. Since PL SLadj did not demonstrate a consistent association with age, subsequent analyses focused on TL SLadj as the primary dependent variable.

Associations between owner-reported cognitive impairment and stride length were explored using linear mixed-effects models with each dog's study ID as a random effect to account for repeated observations within individuals. Fixed effects included CADES total score and the age at visit. To assess whether observed associations were independent of pain and osteoarthritis burden, additional models included CBPI total scores as covariates.

Model assumptions were evaluated using residual diagnostics. Results are reported as regression coefficients with 95% confidence intervals. Statistical significance was set at *p* ≤ 0.05. Analyses were performed using JMP Student Edition 18 (SAS Institute Inc., Cary, NC).

## 3 Results

### 3.1 Population and reliability of stride length measures

The study population included 88 dogs, consisting of 56 spayed females (63.6%), 30 castrated males (34.1%), 1 intact male (1.1%), and 1 intact female (1.1%). Dogs were enrolled at a mean age of 12.7 ± 1.9 years (range 8.6–18.8 years). Thirty-two dogs (36.4%) were mixed breed, and 56 (63.6%) were purebred, with eight Labrador Retrievers, four Beagles, three Golden Retrievers, three Shih Tzus, two Border Collies, two Dachshunds, two Siberian Huskies, two Basset Hounds, and two Jack Russell Terriers. There was one of each of these breeds: Australian Cattle Dog, Australian Shepherd, Bernese Mountain Dog, Border Terrier, Brittany, Brussels Griffon, Cairn Terrier, Cardigan Welsh Corgi, Chihuahua, Chow Chow, Cocker Spaniel, Foxhound, German Shorthair Pointer, Great Dane, Irish Setter, Maltese, Miniature American Shepherd, Pembroke Welsh Corgi, Plott Hound, Pomeranian. Additional information on dogs' height and weight is provided in Table 1.

**Table 1:** Descriptive characteristics of the study population, stride length measures and gait speed.

Variable	Mean ±SD	Median (IQR)	Range
Age (years)	13.23 ± 1.76	13.3 (11.9–14.4)	8.6–18.8
Height (cm)	49.5 ± 12.6	51.0 (40.0–60.0)	21–89
Weight (kg)	19.9 ± 9.7	20.0 (10.7–26.4)	3.4–54.1
Mean TL SL	0.53 ± 0.13	0.55 (0.42–0.63)	0.22–0.87
Mean PL SL	0.54 ± 0.13	0.56 (0.43–0.63)	0.23–0.96
Mean TL SLadj	1.08 ± 0.18	1.07 (0.97–1.17)	0.67–1.86
Mean PL SLadj	1.11 ± 0.17	1.09 (1.00–1.20)	0.69–1.86
Gait speed (m/s)	0.79 ± 0.19	0.77 (0.65–0.90)	0.3–1.46

Data are reported as mean ± SD, median (interquartile range), and range.

Intra-observer reliability was high with ICC (1, 3) = 0.976, 95% CI: 0.952–0.988), based on a subset of 36 measurements; indicating strong consistency of repeated measurements by the same observer. Interobserver reliability was also high with ICC (1, 2) = 0.989 (95% CI: 0.972–0.995) based on a subset of 20 measurements; demonstrating strong agreement between 2 independent observers.

TL SLadj and PL SLadj were not identical on every gait evaluation. Although many dogs had equal stride lengths between thoracic and pelvic limbs, others showed small differences between their mean SLadj in thoracic and pelvic limbs.

Descriptive statistics for thoracic and pelvic limb stride length, with and without height adjustment, are summarized in Table 1.

### 3.2 Association between stride length, body weight and age

**Figure 1:**
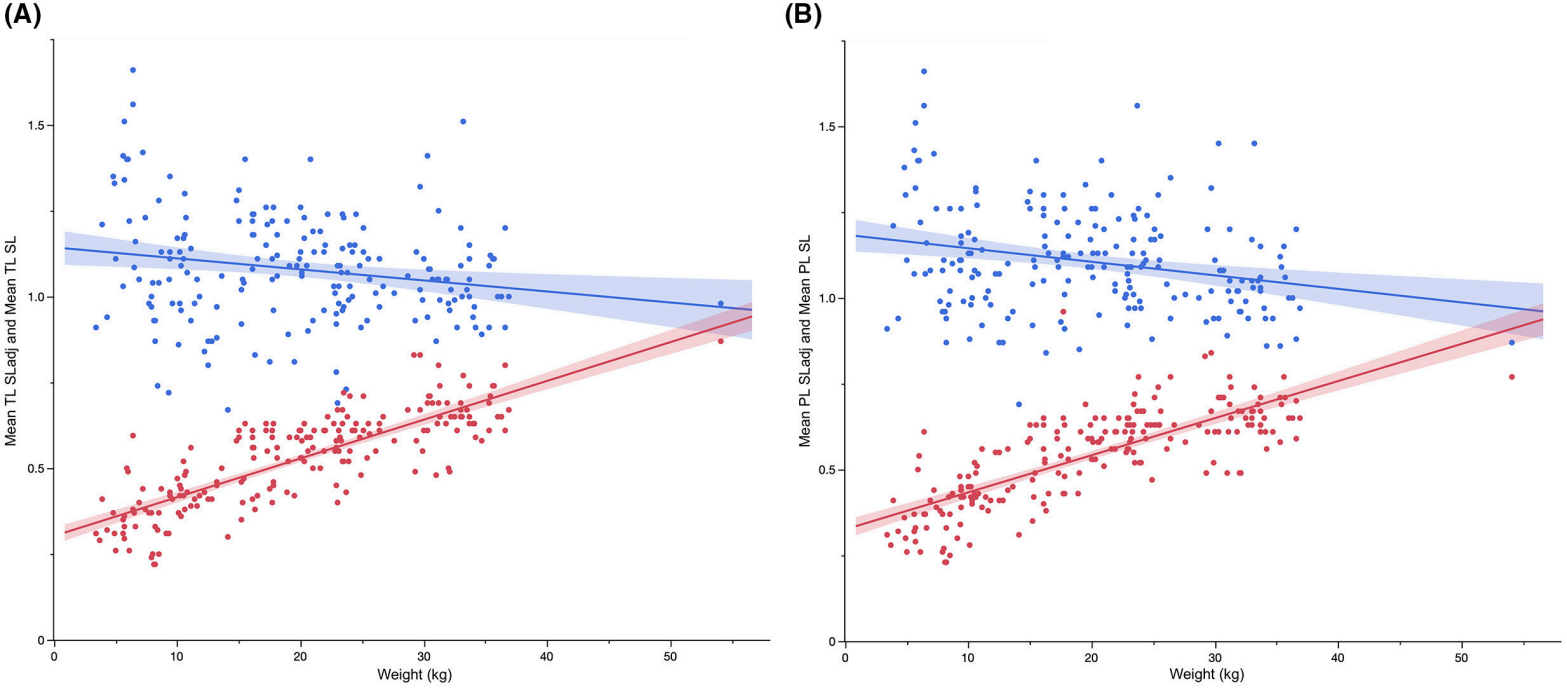
Relationship between body weight and stride length before and after height adjustment. **(A)** Thoracic limbs (TL) and **(B)** pelvic limbs (PL). Scatter plots illustrate the association between body weight and mean unadjusted stride length (SL, red) and mean height-adjusted stride length (SLadj, blue). Unadjusted stride length increased markedly with body weight for both limb pairs, whereas height-adjusted stride length showed a substantially attenuated relationship with body weight. Solid lines represent linear regression fits with shaded bands indicating 95% confidence intervals. TL, thoracic limb; PL, pelvic limb; SL, stride length; SLadj, height-adjusted stride length.

Body weight was strongly associated with unadjusted stride length for both thoracic and pelvic limbs (Figures 1A, B). For thoracic limbs, body weight explained 66% of the variance in stride length (β = 0.011 per kg, *R*^2^ = 0.66, *p* < 0.0001), while for pelvic limbs it explained 58% of the variance (β = 0.010 per kg, *R*^2^ = 0.58, *p* < 0.0001). In contrast, after adjusting for height, the relationship between body weight and height-adjusted stride length was attenuated. For thoracic limbs, body weight explained 4.5% of the variance in SLadj (β = −0.0038 per kg, *R*^2^ = 0.045, *p* = 0.0025), and for pelvic limbs 6.9% (β = −0.0045 per kg, *R*^2^ = 0.069, *p* = 0.0001).

**Figure 2:**
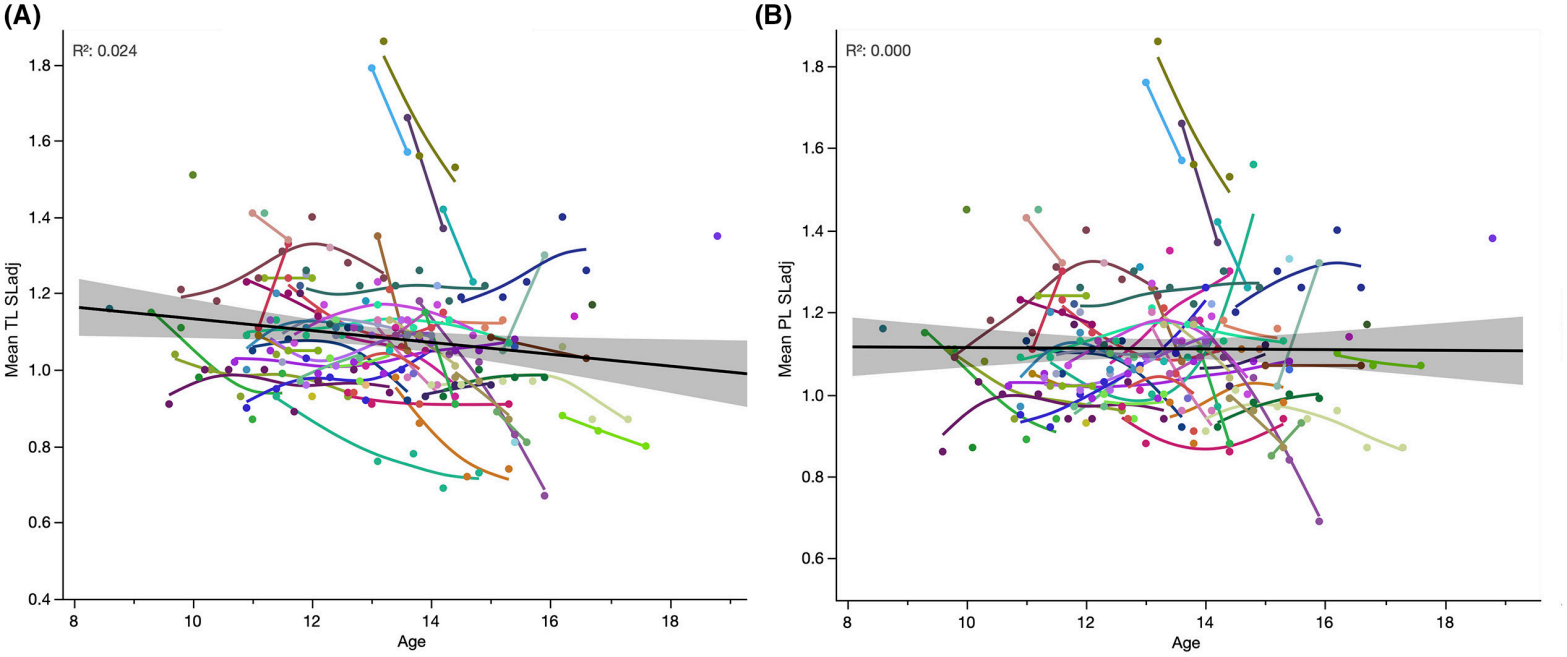
Longitudinal association between age and height-adjusted stride length. The plots illustrate the relationship between age and mean height-adjusted stride length (SLadj) for **(A)** thoracic limbs (TL) and **(B)** pelvic limbs (PL). Individual dogs' trajectories are represented using different colors and the whole model linear regression fits are represented with the black line with shaded bands indicating 95% confidence intervals shown for visualization purposes only. Thoracic limb SLadj demonstrated a significant decline with increasing age, whereas pelvic limb SLadj showed no significant age-related trend. Formal statistical inference was performed using linear mixed-effects models as described in the methods. TL, thoracic limb; PL, pelvic limb; SLadj, height-adjusted stride length.

A significant association with age was observed for mean TL SLadj, whereas no significant association was identified for mean PL SLadj (Figures 2A, B). Longitudinally, TL SLadj showed a relatively consistent decline with advancing age across dogs. In contrast, changes in PL SLadj were inconsistent, with stride length increasing in some dogs and decreasing in others over the study visits, resulting in no clear directional trend at the population level. Therefore, mean TL SLadj was prioritized for subsequent analyses. Across study visits, using a univariate mixed-effects model with age as fixed effect and dog study ID as random effect, the mean TL SLadj decreased significantly with age (β = −0.014975, 95% CI −0.026577 to −0.003373, *p* = 0.0117).

Further analysis in a multivariate model that included limb, age and their interaction identified a significant age-by-limb interaction (β = 0.0147, *p* = 0.0044), indicating that the association between age and height-adjusted stride length differed between thoracic and pelvic limbs. Thoracic limb height-adjusted stride length decreased with age (β = −0.0129), whereas pelvic limb height-adjusted stride length showed little change over time (β = 0.0018).

### 3.3 Association between stride length, cognitive and pain scores

**Figure 3:**
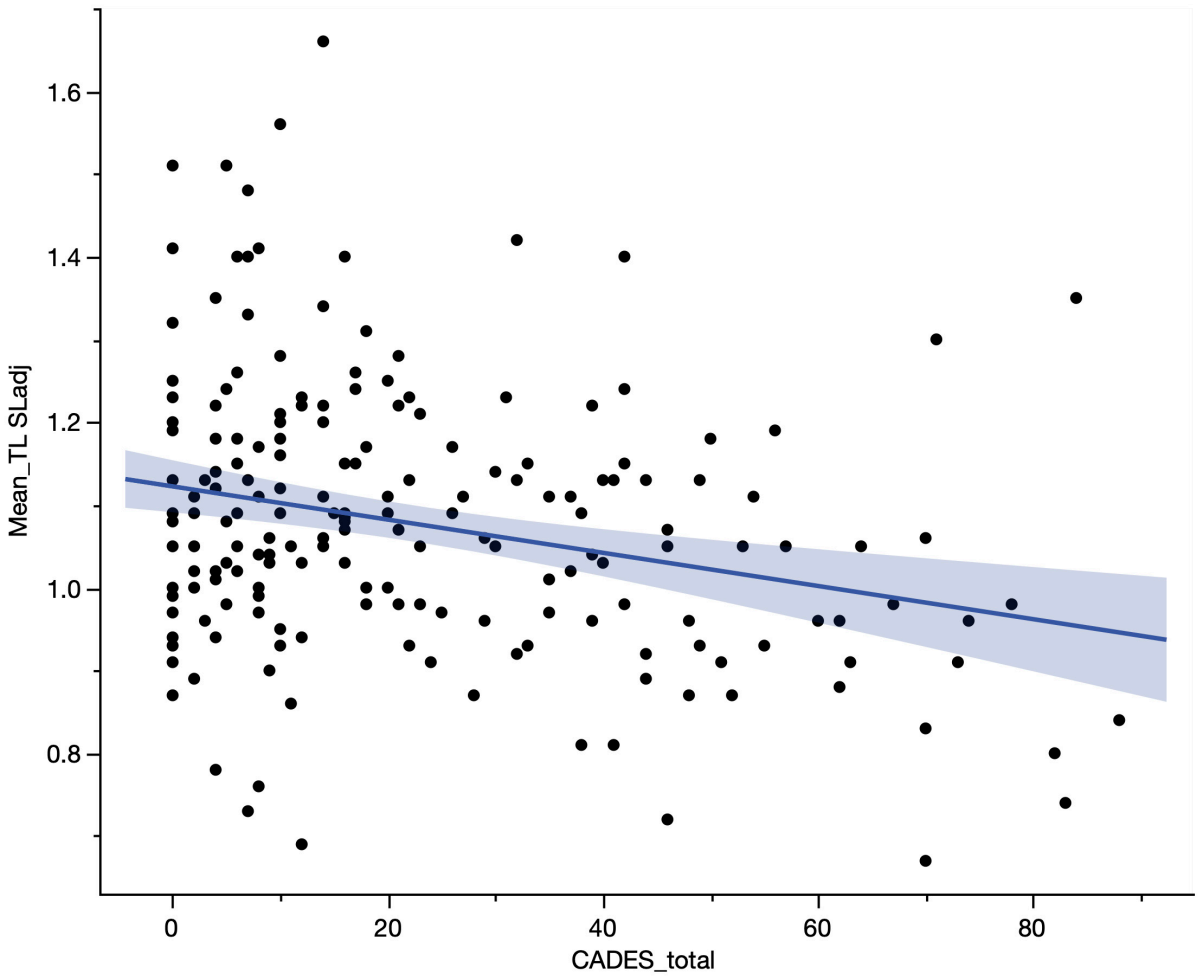
Association between owner-reported cognitive impairment and thoracic limb height-adjusted stride length. Scatter plot illustrating the relationship between CADES total score and mean thoracic limb height-adjusted stride length (TL SLadj). Solid line represents the linear regression fit with shaded band indicating the 95% confidence interval. Increasing CADES scores were associated with shorter TL SLadj. CADES, Canine Dementia Scale; TL, thoracic limb; SLadj, height-adjusted stride length.

In a univariate mixed-effects model, there was a negative association between mean TL SLadj and CADES score (Figure 3). Higher CADES scores, representing more severe cognitive decline, were associated with lower mean TL SLadj (β = −0.001652, 95% CI −0.002515 to −0.000789, *p* = 0.0002). After adjusting for age in a multivariate mixed-effects model, CADES score remained significantly associated with mean TL SLadj (β = −0.001476, 95% CI −0.0025 to −0.000452, *p* = 0.0050), while age was not significant in that model (β = −0.004388, SE 0.0069464, 95% CI −0.018092 to 0.0093154, *p* = 0.5283). To further investigate the specific gait characteristics associated with cognitive status, a multivariate model was constructed with CADES score as the dependent variable and both mean TL SLadj and gait speed as fixed effects. In this model, mean TL SLadj remained a significant predictor of cognitive score (β = −26.7, 5% CI:−48.87 to−4.51, *p* = 0.0187), while gait speed was not significant.

To determine whether the observed age-related shortening of the mean TL SLadj could be explained by progressive osteoarthritis-associated pain, the relationship between the mean TL SLadj and Canine Brief Pain Inventory (CBPI) score was evaluated using a multivariate mixed-effects model, with age as a fixed effect. CBPI total score was independently associated with shorter stride length (β = −0.001241, 95% CI −0.002412 to −0.000070, *p* = 0.0379), while age remained non-significant. When the CBPI total score was included in the age-adjusted multivariate mixed-effects model with CADES score and mean TL SLadj, the CADES score remained negatively associated with stride length (β = −0.001281, 95% CI −0.002375 to −0.000187, *p* = 0.0221).

## 4 Discussion

In this longitudinal study of aging companion dogs, owner-reported cognitive decline was associated with shorter mean thoracic limb stride length, adjusted for height. Higher CADES scores were associated with reduced stride length, and this relationship persisted after adjustment for age and owner-reported pain impact on mobility as measured by the CBPI score. Together, these findings indicate that owner-perceived cognitive decline, particularly as captured by the CADES questionnaire, is reflected in measurable changes in gait characteristics in aging dogs. Notably, although CADES scores were correlated with age, the association between CADES and stride length remained after accounting for chronological age, suggesting that cognitive status captures aspects of functional decline not fully explained by age alone.

The relationship between cognition and gait has been well described in human aging and dementia research (1, 2). In older adults, shorter stride length and altered spatial gait parameters are associated with cognitive impairment, increased fall risk, and progression of Alzheimer-type dementia (5, 6). Studies of individuals with dementia have demonstrated that worsening cognitive decline is accompanied by reductions in stride length, even when controlling for musculoskeletal factors (6). Gait abnormalities in these populations are thought to reflect impaired central regulation of movement, including deficits in attention, executive control, and motor planning (1, 4).

Mobility impairment is increasingly recognized as a clinically relevant component of aging and cognitive decline in dogs, with owners reporting unsteadiness, swaying, and falls alongside behavioral changes (8, 16). However, whether these mobility changes reflect cognitive dysfunction itself or are secondary to pain and orthopedic disease remains unclear. Our findings help to clarify this relationship by demonstrating an association between thoracic limb stride length and owner-reported cognitive decline that persisted after adjustment for age and owner-reported pain. Our findings in aging dogs parallel human observations, supporting the notion that spatial gait parameters may capture aspects of neurodegenerative change beyond simple movement slowing.

In a secondary analysis, we evaluated the relative contributions of TL SLadj and on-leash gait speed to cognitive status. When both were included in a multivariate model, mean TL SLadj remained significantly associated with CADES scores, whereas gait speed did not. This suggests that while these measures are biomechanically related, height-adjusted stride length might capture a distinctive spatial signal of cognitive impairment that is not fully explained by raw walking velocity.

Even though we measured PL SL and adjusted the measurements to dogs' withers height, no consistent age-related pattern was observed in this population. This was supported by a significant limb-by-age interaction in the mixed-effects model, indicating that thoracic and pelvic limb stride lengths do not change in parallel with aging. Pelvic limb gait characteristics in aging dogs are frequently affected by coxofemoral osteoarthritis, stifle joint diseases, and lumbosacral pathology (13, 25). As a result, PL SL may capture overlapping effects of orthopedic disease and central neurologic aging, making it more difficult to isolate changes specifically associated with cognitive decline. In addition to these orthopedic influences, locomotion is generated by a central pattern generator that produces rhythmic motor activity through modular, limb- and joint-specific neural circuits (26). These centrally generated patterns are continuously modulated by sensory input, including proprioceptive, visual, and vestibular information required for postural control and navigation (27). Thoracic limbs play a key role in braking and postural stabilization, whereas pelvic limbs primarily contribute to propulsion (28, 29). As a result, thoracic limb movement may be more sensitive to alterations in integrated sensorimotor control, while pelvic limb movement may be more influenced by rhythmic locomotor patterning and musculoskeletal factors. Therefore, we focused the subsequent analyses on the mean TL SLadj.

From a clinical perspective, stride length offers several practical advantages as a mobility metric in geriatric dogs. It can be derived from brief, standardized walkway assessments using routine video recordings, normalized to body size, and averaged across passes to improve reliability (30–32). Unlike gait speed alone, stride length reflects gait organization and central motor control, which rely on higher-order neural processes known to be affected by cognitive decline (33–35). The observed association between CADES score and stride length, independent of age and owner-reported pain, suggests that stride length may provide complementary information to owner questionnaires when monitoring functional decline in aging dogs. Although the estimated change per 1-point increase in CADES was small, it became more clinically interpretable across broader score differences. Based on the model coefficient, a 10-point increase in CADES corresponded to an approximate 1.2% reduction in mean TL SLadj. While this effect size is small and not sufficient for use as an individual diagnostic tool, it is consistent with the concept that mobility measures may serve as early indicators of functional decline along a broader aging trajectory rather than as standalone clinical tests. As such, stride length is more likely to serve as an adjunctive marker of an individual's clinical trajectory, especially when assessed longitudinally and alongside cognitive assessments.

The association between the CBPI score and the mean TL SLadj suggests that pain-related functional impairment contributes to overall gait performance in aging dogs. Furthermore, the association between cognitive impairment, as measured by the CADES score, and the mean TL SLadj persisted after accounting for owner-reported pain, indicating that reduced mean TL SLadj cannot be explained entirely by osteoarthritis-associated pain. Together, these findings highlight the multifactorial nature of shortening of stride length and mobility decline in older dogs, with both cognitive status and pain-related functional limitation influencing gait performance. These results underscore the importance of incorporating stride length measures in the geriatric evaluation of aging dogs and aim for a multimodal approach that integrates cognitive, mobility, and pain domains to provide a more thorough picture of their health.

Several limitations should be acknowledged. Cognitive dysfunction and pain were assessed using owner-reported questionnaires, rather than clinical diagnosis, to capture the lived experience of the dogs. However, these questionnaires do not establish specific diagnoses, rather they capture functional behavioral changes in the home. Furthermore, while the longitudinal design and mixed-effects modeling accounted for repeated measures within dogs, causal relationships between cognitive decline and gait changes cannot be inferred. Also, despite normalizing the stride length to withers height, differences in conformation and gait mechanics across breeds may still influence stride characteristics that were not explicitly modeled in our analysis. Moreover, inclusion required dogs to be ambulatory and tolerant of standardized mobility assessments, which may have resulted in underrepresentation of dogs with severe mobility impairment, advanced neurologic disease, or behavioral limitations. As a result, associations observed in this cohort do not fully reflect gait characteristics in more severely affected aging dogs.

Reliability estimates for stride length measurements were high. However, these were derived from a subset of the total population. While the number of individual measurements (n=32 for intra-observer; n=20 for interobserver) provides confidence in the technical repeatability of the method, these estimates should be considered preliminary. Future studies with larger cohorts across diverse walking environments would further validate the scalability of this metric.

## 5 Conclusion

This study demonstrates that owner-reported cognitive decline, as measured by CADES, is associated with reduced stride length in aging companion dogs. This relationship persisted after adjusting for age and owner-reported pain, indicating that CADES could capture variations in gait that are not explained by age alone. These findings support the clinical relevance of stride length as an objective, scalable mobility measure that reflects functional changes associated with cognitive aging. Incorporating stride-length assessment into routine geriatric evaluations may improve the detection and monitoring of functional decline in older dogs and support more informed discussions of quality of life and management strategies.

## Data Availability

The raw data supporting the conclusions of this article will be made available by the authors, without undue reservation.
